# Akutes Nierenversagen nach SARS-CoV-2-Infektion

**DOI:** 10.1007/s11560-022-00593-8

**Published:** 2022-06-24

**Authors:** Alexandre Klopp, Tobias B. Huber, Thorsten Wiech, Ulrich O. Wenzel

**Affiliations:** 1 III. Medizinische Klinik und Poliklinik, Universitätsklinikum Hamburg-Eppendorf, Martinistr. 52, 20246 Hamburg, Deutschland; 2 Institut für Pathologie, Sektion Nierenpathologie, Universitätsklinikum Hamburg-Eppendorf, Hamburg, Deutschland

## Anamnese

Eine 55-jährige Patientin wurde vom behandelnden Nephrologen zur weiteren Abklärung eines akuten Nierenversagens unklarer Ätiologie eingewiesen. Das Serumkreatinin bei Aufnahme betrug 1,78 mg/dl. 2 Monate zuvor lag das Kreatinin noch bei 0,85 mg/dl. Anamnestisch beschrieb die Patientin lediglich eine vermehrte Abgeschlagenheit, die seit 2 Monaten bestünde und am ehesten auf eine kürzlich durchgemachte SARS-CoV-2(„severe acute respiratory syndrome coronavirus 2“)-Infektion zurückzuführen sei. Als Vorerkrankung bestünde nur ein Glaukom, das sie mit Augentropfen therapiere, ansonsten nehme sie keine Medikamente ein.

## Klinische Befunde

Die initiale laborchemische Untersuchung war bis auf das vorbeschriebene erhöhte Serumkreatinin sowie eine milde mikrozytäre Anämie (Hämoglobin: 11,6 g/dl) absolut unauffällig. In der Urindiagnostik fand sich eine Albuminurie von 209 mg pro g Kreatinin. Das Urinsediment wies vereinzelt dysmorphe Erythrozyten auf. In der Ultraschalluntersuchung zeigten sich beidseits regelrecht konfigurierte Nieren mit einer echoreich verdichteten Parenchymstruktur.

## Weiteres Prozedere

**Figure 1:**
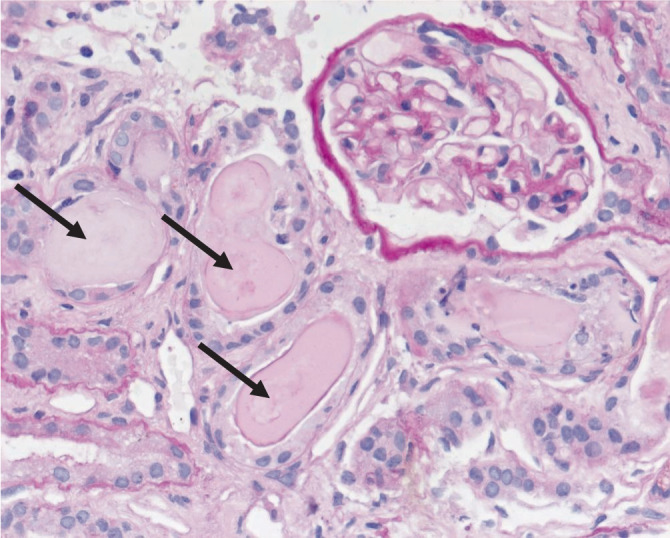

Bei unklarer Ätiologie des Nierenversagens entschieden wir uns zur weiteren Diagnostik für eine Nierenbiopsie. Wir erwarteten eine Schädigung der Niere im Zusammenhang mit der kürzlich durchgemachten SARS-CoV-2-Infektion. Renale Beteiligung im Rahmen einer SARS-CoV-2-Infektion wurde von uns schon früh beschrieben [1]. Die Lichtmikroskopie ist in Abb. 1 dargestellt.

## Wie lautet Ihre Diagnose?

## Diagnose

**Figure 2:**
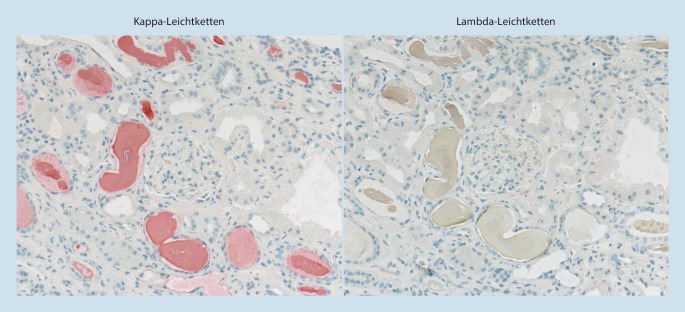

In der histopathologischen Aufarbeitung der Nierenbiopsie zeigte sich das typische Bild einer Plasmozytomniere mit immunhistochemischer Positivität intratubulärer Eiweißzylinder für Kappa-Leichtketten und Negativität für Lambda-Leichtketten (Leichtketten-Cast-Nephropathie; Abb. 2). Der Biopsiebefund ließ sich in den durchgeführten laborchemischen Untersuchungen bestätigen, wo sich unter anderem in der Immunfixation eine monoklonale Gammopathie vom Typ IgG(Immunglobulin G)-kappa offenbarte. Die definitive Diagnosestellung erfolgte anschließend per Knochenmarkpunktion. In Zusammenschau der laborchemischen und histopathologischen Befunde ergab sich also die Diagnose eines akuten Nierenversagens auf dem Boden eines erstdiagnostizierten multiplen Myeloms. Bei erneutem Nachfragen korrigierte die Patientin Ihre Anamnese und schilderte, dass die verminderte Leistungsfähigkeit schon vor der Infektion aufgetreten sei.

## Therapie und Verlauf

Bei Vorliegen einer relevanten Endorganschädigung (in diesem Fall der Nieren) waren die von der International Myeloma Working Group definierten CRAB(„increased plasma calcium level, renal insufficiency, anemia, bone lesions“)-Kriterien [2] erfüllt und somit die Indikation einer gegen das multiple Myelom gerichteten Chemotherapie gegeben. Es erfolgte eine Induktionstherapie mittels Daratumumab, Bortezomib, Dexamethason und Thalidomid.

## Diskussion

Im Rahmen von klinischen Entscheidungen arbeiten wir mit Mustererkennung. Nicht zuletzt auch zur Schonung der Ressourcen sollte die Diagnostik gemäß Anamnese und den vorliegenden Symptomen durchgeführt werden. Im vorliegenden Fall gab es eine komplett unauffällige Anamnese bis auf eine durchgemachte SARS-CoV-2-Infektion sowie eine von der Patientin geschilderte Schwäche und Abgeschlagenheit seit der Infektion. Nicht zuletzt durch hochrangige Publikationen, die eine Beteiligung der Niere bei SARS-CoV-2-Infektionen zeigen, hatten wir die Erwartungshaltung, eine Erkrankung zu finden, die durch die Infektion verursacht oder zumindest ausgelöst bzw. getriggert wurde. Der Befund einer Plasmozytomniere war daher unerwartet.

Eine renale Beteiligung ist eine häufige Komplikation des multiplen Myeloms. Zum Zeitpunkt der Diagnose weisen 20–50 % der Myelompatienten bereits Zeichen einer Nierenschädigung auf – ob chronisch oder akut [3]. Dieser Fall unterstreicht die Wichtigkeit einer umfassenden ätiologischen Abklärung eines akuten Nierenversagens. Auch bei fehlenden klinischen Hinweisen sowie weitgehend unauffälligem laborchemischem Befund sollte bei akutem Nierenversagen unklarer Ätiologie auch immer an ein multiples Myelom als Grunderkrankung gedacht werden [4]. Ob es sinnvoll ist, bei jeder unklaren Nierenerkrankung Leichtketten in Serum und Urin zu bestimmen, muss bei knapper werdenden Ressourcen kritisch diskutiert werden.

**Diagnose:** Cast-Nephropathie

Dieser Fall veranschaulicht den Stellenwert einer Nierenbiopsie in der Abklärung eines akuten Nierenversagens, die oft im Gegensatz zu aufwendigen laborchemischen Untersuchungen eine gezielte, kosteneffiziente und therapierelevante Diagnosestellung gewährleistet und zusätzlich bereits eine Aussage über die Prognose der Nierenschädigung ermöglicht. Der Fall zeigt aber auch, dass Mustererkennung und Erwartungshaltung in der Nephrologie täuschen und die korrekte Diagnosevorstellung verzögern können.

## Fazit für die Praxis

- Mustererkennung und Erwartungshaltung können täuschen und die korrekte Diagnosestellung verzögern.
- Ein multiples Myelom sollte bei jedem Nierenversagen unklarer Ätiologie angedacht werden, auch bei initial weitgehend unauffälligen klinisch-serologischen Befunden.
- Die Nierenbiopsie mit anschließender histopathologischer Aufarbeitung bleibt ein zentraler und wichtiger Bestandteil in der Ursachenforschung eines akuten Nierenversagens.
